# Microarray-based identification of antigenic variants of foot-and-mouth disease virus: a bioinformatics quality assessment

**DOI:** 10.1186/1471-2164-7-117

**Published:** 2006-05-18

**Authors:** Verónica Martín, Celia Perales, David Abia, Angel R Ortíz, Esteban Domingo, Carlos Briones

**Affiliations:** 1 Centro de Biología Molecular "Severo Ochoa", Universidad Autónoma de Madrid, Cantoblanco, 28049, Madrid, Spain; 2 Bioinformatics Unit, Centro de Biología Molecular "Severo Ochoa", Universidad Autónoma de Madrid, Cantoblanco, 28049, Madrid, Spain; 3 Centro de Astobiología (CSIC-INTA), Torrejón de Ardoz, 28850, Madrid, Spain

## Abstract

**Background:**

The evolution of viral quasispecies can influence viral pathogenesis and the response to antiviral treatments. Mutant clouds in infected organisms represent the first stage in the genetic and antigenic diversification of RNA viruses, such as foot and mouth disease virus (FMDV), an important animal pathogen. Antigenic variants of FMDV have been classically diagnosed by immunological or RT-PCR-based methods. DNA microarrays are becoming increasingly useful for the analysis of gene expression and single nucleotide polymorphisms (SNPs). Recently, a FMDV microarray was described to detect simultaneously the seven FMDV serotypes. These results encourage the development of new oligonucleotide microarrays to probe the fine genetic and antigenic composition of FMDV for diagnosis, vaccine design, and to gain insight into the molecular epidemiology of this pathogen.

**Results:**

A FMDV microarray was designed and optimized to detect SNPs at a major antigenic site of the virus. A screening of point mutants of the genomic region encoding antigenic site A of FMDV C-S8c1 was achieved. The hybridization pattern of a mutant includes specific positive and negative signals as well as crosshybridization signals, which are of different intensity depending on the thermodynamic stability of each probe-target pair. Moreover, an array bioinformatic classification method was developed to evaluate the hybridization signals. This statistical analysis shows that the procedure allows a very accurate classification per variant genome.

**Conclusion:**

A specific approach based on a microarray platform aimed at distinguishing point mutants within an important determinant of antigenicity and host cell tropism, namely the G-H loop of capsid protein VP1, was developed. The procedure is of general applicability as a test for specificity and discriminatory power of microarray-based diagnostic procedures using multiple oligonucleotide probes.

## Background

The control of diseases associated with highly variable RNA viruses requires close monitoring of the variant virus types that periodically dominate in viral populations. This is due to high mutation rates, quasispecies dynamics and population bottlenecks that often accompany virus transmission [reviewed in [1]]. Indeed, RNA viruses replicate with mutation rates in the range of 10^-3 ^to 10^-5 ^substitutions per nucleotide copied [2,3]. As a consequence, RNA virus populations consist of complex and dynamic distributions of related genomes termed viral quasispecies [4,5]. Viral quasispecies can influence viral pathogenesis [6-8], and the response to antiviral treatments [9]. Mutant clouds in infected organisms represent the first stage in the natural genetic and antigenic diversification of viruses [8,10]. A consequence which is relevant to viral diagnosis and surveillance is that a transmission bottleneck may result in the establishment in the recipient host of one (or few) variant(s) sampled from the mutant cloud that replicates in the infected donor. Therefore, methodology to discern among minor variants of the same viral genotype or serotype is essential for epidemiological surveillance and the planning of disease control strategies.

An important animal pathogen which participates of quasispecies dynamics, transmission bottlenecks, and the potential for rapid evolution is foot-and-mouth disease virus (FMDV), the etiological agent of the economically most devastating disease of farm animals [recent reviews in [11]]. FMDV is an aphthovirus of the family *Picornaviridae*, whose genome is a single stranded RNA of about 8200 nucleotides, of positive polarity, replicated by a virus-coded RNA-dependent RNA polymerase, devoid of a proofreading-repair activity [12]. The antigenic variation of FMDV is a direct consequence of its genetic variation during natural infections, confirmed by many experiments *in vivo *and in cell culture [11,13]. Inactivated virus vaccines are used to control FMD, but their efficacy is limited by the antigenic variation of the virus [11]. The antigenic diversity of FMDV is reflected in the occurrence of seven serotypes (A, O, C, Asia1, SAT1, SAT2, SAT3), and multiple subtypes and variants that defy classification due to the continuous recognition of mutant forms in replicating FMDV quasispecies [14]. In vaccination-challenge experiments no cross-protection is observed among representatives of a different serotype, and only partial protection among some subtypes and variants [11]. Therefore, continuous monitoring of circulating antigenic forms is required to prepare vaccines whose antigenic composition matches that of the circulating virus [11].

Antigenic variants of FMDV have been classically diagnosed by immunological methods (complement fixation, ELISA, neutralization of infectivity) [review in [15]]. Recently, several methods based on reverse transcription-PCR (RT-PCR) amplification have been adapted to the diagnosis of FMDV [16]. Some of these methods can be applied without the need to grow the virus in cell culture. More recently, a FMD DNA chip containing 155 oligonucleotide probes to detect simultaneously the seven FMDV serotypes has been described [17]. Several studies have documented that long oligonucleotide DNA microarrays can detect simultaneously many viral pathogens [18]. Multiple oligoprobes were used to characterize the heterogeneous composition and recombination forms of human poliovirus [19]. These results encourage the development of a new microarray-based approach to probe the fine genetic and antigenic composition of FMDV for diagnosis, vaccine design, and to gain insight into the molecular epidemiology of this pathogen.

**Figure 1:**
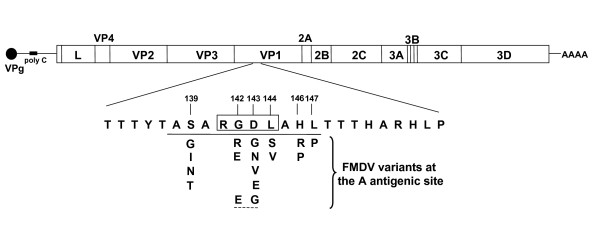
**Scheme of the FMDV genome and repertoire of mAb SD6-escape mutants included in the microarray**. Top: C-S8c1 genome (8115 nucleotides, excluding homopolymeric tracts); boxes indicate encoded proteins and lines indicate regulatory regions (not drawn to scale); the filled circle represents protein VPg covalently linked to the 5' end of the RNA, and AAAA represents the 3'-terminal polyadenylate tract. Below the genome, the amino acid sequence of the G-H loop of the VP1 protein (amino acids 133 to 156) is shown; the epitope defined by mAb SD6 is underlined. The sequence RGDL involved in integrin recognition is boxed. Below the mAb SD6 epitope sequence, the amino acid replacements found in individual biological FMDV clones isolated as mAb SD6-escape mutants whose corresponding mutations have been analysed in the microarray, are indicated. The discontinuous line at the bottom indicates a double mutant. Based in [11, 13, 44] and references therein.

A major antigenic site of FMDV (termed site A) is located at the mobile, exposed G-H loop of capsid protein VP1 [13,20,21]. This loop includes several epitopes involved in binding of neutralizing antibodies, as well as an Arg-Gly-Asp (RGD) triplet that participates in recognition of integrin receptors [21,22]. The overlap of residues involved in receptor recognition and antibody binding implies that variations at the G-H loop of VP1 can have consequences both for the antigenic behavior of the virus and its host range [23,24]. For FMDV of serotype C multiple variants at the epitopes located within antigenic site A were documented among natural populations of the virus. Furthermore, studies in cell culture have shown that FMDV can evolve towards variants with altered RGD that display a remarkable expansion of host cell tropism [25]. The several biological implications of the G-H loop of VP1 prompted us to develop a DNA oligonucleotide microarray to probe multiple genetic variants of FMDV, around VP1 residues 139 to 147 (Figure 1). We report assay conditions that have been optimized to detect the presence of several point mutants at this major antigenic site of FMDV, and develop a support vector machine (SVM)-based procedure to automatize sample classification hybridization intensities and to set up limits for reliable diagnosis.

## Results

### Specificity and sensitivity optimization of FMDV microarray

**Figure 2:**
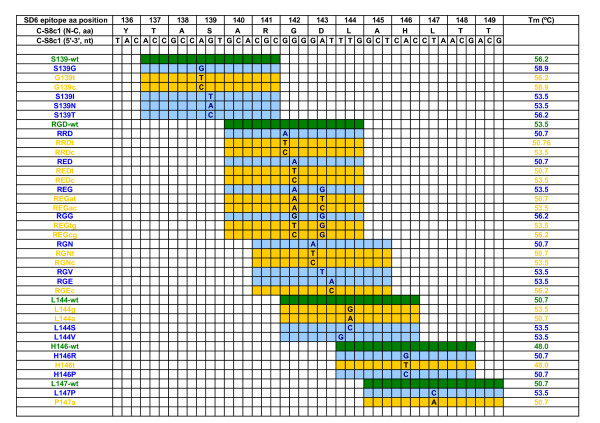
**Oligonucleotides printed on the microarray for the screening of SD6 epitope C-S8c1 FMDV variants**. Top: Amino acid and nucleotide sequence encoding the epitope defined by mAb SD6 in FMDV C-S8c1 VP1, between codons 136 and 149. The column on the left shows the names of the oligonucleotides used in this work depicted as colored boxes on the right; the boxes in green represent oligonucleotide sequences identical to the wild type nucleotide sequence written at the top; the boxes in blue represent the oligonucleotides with a sequence corresponding to the different mAb SD6-scape mutants; the boxes in yellow represent oligonucleotide sequences used as negative hybridization controls. Colored, empty boxes include the wild type nucleotide. The mutations are specified in the corresponding position. The enquired position is located at the center of the oligonucleotide. The column on the right gives the predicted Tm value for each oligonucleotide, calculated according to Tm = 69.5 + 0.41 × (X%G+C)-650/total nucleotide number. The origin of the different mutations analysed is given in Methods and in Figure 1.

In a first approach, 8 DNA oligonucleotides were designed for the set up of an FMDV microarray. They represent RNA sequences encoding the G-H (VP1) loop of C-S8c1 FMDV. Two variants (encoding RGD and RED at VP1 positions 141–143) (Figures 1 and 2) of FMDV were initially tested. A microarray with both FMDV variants was printed to analyze the influence of long (15-mer) versus short (11-mer) oligonucleotides, the presence or absence of (dT)_15 _spacers, and the oligonucleotide concentration. A number of conclusions were drawn from the results (not shown). First, the hybridization signals were weaker with oligonucleotides of 11 residues than with oligonucleotides of 15 residues. We have not assessed oligonucleotides longer than 15 residues because they are more likely to accommodate, without destabilization of the helical duplex, a single nucleotide mismatch at a central position [26]. The second observation was that oligonucleotides linked through a (dT)_15 _track hybridized more efficiently than those without the track in agreement with previous results [27]. Third, the experiments indicated that the amount of oligonucleotide attached at concentrations between 5 and 50 μM was not limiting for detection of fluorescent DNAs. We chose the highest concentration tested for the standard protocol. Preliminary experiments showed also that hybridization solutions including 50% formamide resulted in poor sensitivity, and that the Unyhib solution (Arrayit) produced results comparable to those obtained with the hybridization solution described in Methods. To generate labeled targets, two different systems were used: direct labeling with Cy3-dUTP and Cy5-dUTP, and indirect labeling with Alexa Fluor 647; the latter proved easier, more reproducible, efficient and yielded targets showing higher stability.

**Figure 3:**
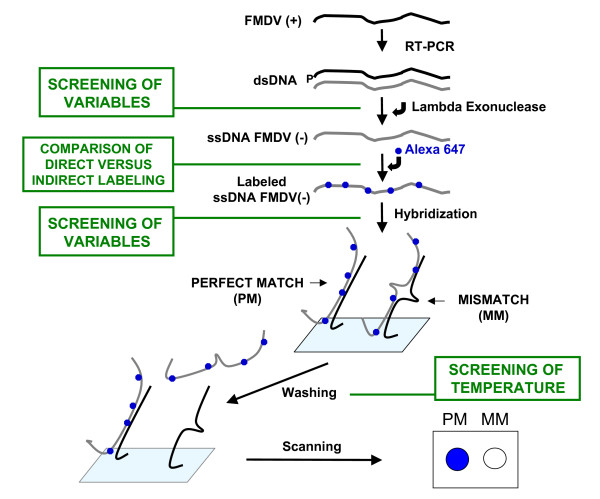
**Scheme of the successive steps from the copying of FMDV genomic RNA to scanning of the microarray**. RT-PCR was performed using primers 1R1L (phosphorylated at its 5'-end) and pUL; their sequences are given in Methods. Green boxes indicate those steps for which a number of variables were tested; details on the results of such variable screening will be provided upon request. The final protocol used for the different steps is detailed in Methods.

A step-wise increase of hybridization temperatures, between 48°C and 62°C, was tested. Low temperatures resulted in poor microarray performance due to high number of false positives. The optimal point mutation discrimination was obtained between 58°C and 60°C. Higher temperatures resulted in a progressive and significant loss of signal. Similar comparisons revealed 45°C as the most adequate temperature for washing the hybridized microarrays. A scheme of the entire procedure with indication of the steps for which variables were screened is depicted in Figure 3.

### Screening of point mutants of the genomic region encoding antigenic site A of FMDV C-S8c1

**Figure 4:**
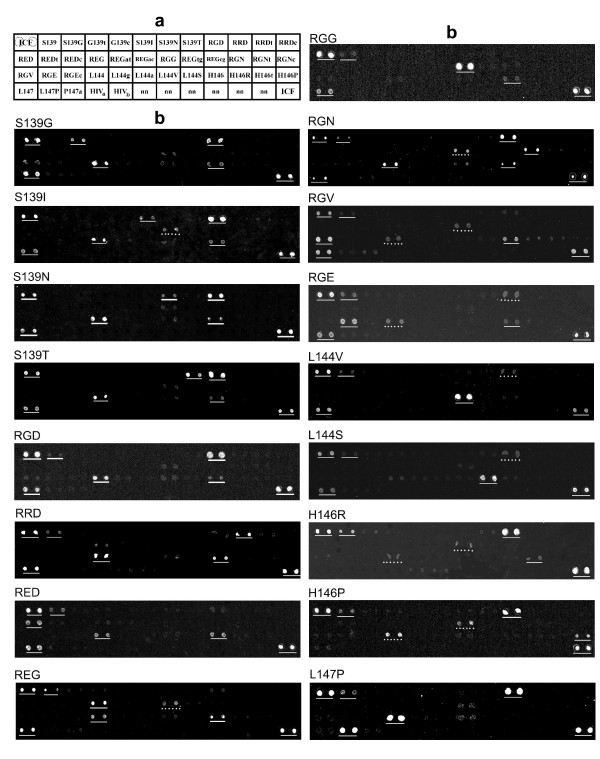
**Display of oligonucleotides on the microarrays and microarray hybridization patterns of 16 mAb SD6-escape mutants of FMDV**. **a**. Forty-one oligonucleotides (50 μM) were spotted in duplicate, as indicated by dotted circles in the top left box of the top left grid; each box in the grid includes the name of a oligonucleotide (sequence given in Figure 2) or negative controls (HIVa, HIVb and nn). Oligonucleotides are distributed in 4 rows and 12 columns. An oligonucleotide representing the conserved sequence 5'-CCTAGGCCGATTCTTCCG-3', C-S8c1 genomic residues 3757 to 3775 was used as a positive control of hybridization (termed ICF, Internal Control of FMDV). The ICF oligonucleotide was printed on the left top and right bottom corners of the grids, as indicated. The pattern was printed four times in each microarray. **b. **Each panel represents a microarray image, given by the Alexa Fluor 647 fluorescence signal, after hybridization, washing and scanning, as detailed in Methods. The distribution of oligonucleotide probes in each array is identical to that given in *a*. The name of the target sequence is written at the left top of each panel; the nucleotide sequence of the different targets is given in Figure 2. Positions expected to give a positive signal (perfect match) are underlined. Positions indicated by a dotted line show a mismatch hybridization but not in the central position. A weak signal due to crosshybridization is expected in these probes.

A total of 11 positions within genomic residues 3616 to 3654 were analyzed by constructing 15-mer oligonucleotides with the queried nucleotide (and a number of negative control mismatched nucleotides) located at position 7 to 11 in each 15-mer (Figure 2). Forty-one oligonucleotides were spotted in duplicate, distributed in 4 rows and 12 columns per grid (Figure 4). A conserved FMDV sequence was used as positive control for the hybridization (ICF). Two unrelated HIV oligonucleotides (HIVa and HIVb) and spots with no nucleotide (nn) were used as negative control. The same pattern containing spots with 15-mers corresponding to the different queried and control mutants, and positive (ICF) and negative (HIV, nn) controls were printed four times per slide.

RT-PCR products obtained with RNA from each of 16 mAb SD6-escape mutants of FMDV as template and primers 5'P-1R1L and pUL, were treated with lambda exonuclease, and labeled with Alexa Fluor 647 as detailed in Methods. The labeled DNA was hybridized in the microarray, as described in Methods.

Five oligonucleotides were designed to identify the wild-type C-S8c1 sequence at the following positions: 139 (S139), 142 and 143 (RGD), 144 (L144), 146 (H146) and 147 (L147). In the RGD panel good signal intensity was obtained at four of the positions tested (Figure 4); only the hybridization with S139 oligonucleotide produced a low signal.

Four mutants at position 139 were tested. Each mutant could be identified due to a high signal in the perfect match probe. No crosshybridization with G139t, G139c or S139 was detected (Figure 4b).

Two point mutants at VP1 position 142, RRD and RED, as well as a double mutant for the 142 and 143 positions (REG), were available for testing. Hybridizations with each mutant generated positive signals with the wild-type oligonucleotides that did not include positions 142 and 143. However, hybridizations were positive with the probe that identified specifically each mutant, but not with the probe that represented the wild-type RGD sequence (Figure 4b).

Position 143 is represented by four SD6-escape mutants: RGG, RGN, RGV and RGE. Each of them, as well as substitutions at position 144 (Figure 4b), produced the expected signal. Substitutions at position 144 were perfectly discriminated with the oligonucleotides designed in the microarray (Figure 4) with a slightly weak signal with the S139 probe. The three mutants analyzed at positions 146 and 147, named H146R, H146P and L147P, showed an adequate signal for specific identification, and no crosshybridization with other probes at the same position.

The results (Figure 4b) indicate a good discrimination between positive and negative signals as well as strong signals in the ICF probe and no signal in any of the negative controls (HIVa HIVb and nn probes), as expected from the perfect match and mismatch hybridization signals, respectively. However, the hybridization pattern of a mutant includes specific negative and positive signals as well as crosshybridization signals, which are of different intensity depending on the hybridization kinetics of each probe and target. Therefore, an array classification method was developed to evaluate the hybridization signals.

### Microarray quantification and quality control of hybridization signals

Procedures for microarray quantification, quality control of hybridization intensities, and data classification were applied to the microarray signals, as described in Methods. Jack-knife tests yielded a class averaged classification accuracy of 98.7 ± 2.4%. Table 1 shows classification accuracy per variant. Most variants are predicted above 95% accuracy. Exceptions include phenotypes RGE1 and RGV, with about 93% prediction accuracy. In order to study the distribution of errors, a confusion matrix is shown in Table 2. The matrix reveals that the small fraction of errors observed shows a systematic distribution. Thus, misclassified RGE samples are systematically classified as S139T samples, while a misclassified RGV sample is classified within the RGD variant, and a misclassified V144 sample is assigned to the RED mutant. Most likely, the observed errors have their origin in hybridization artefacts, and will probably be corrected in future versions of the chip. Nevertheless the achieved accuracy is already satisfactory in all cases for practical applications.

**Table 1:** Summary of the data set and classification results. Average accuracy for each class (Av.class.acc.), i.e, the average of the number of successfully classified samples divided by the number of classified samples in 100 rounds of jack-knife, and its standard deviation (St.dev.class.acc.) are shown for each phenotype. The total number of samples in each class (#samples) is also given.

**Phenotype**	**#samples**	**Av.class.acc.**	**St.dev.class.acc.**
RGD	25	1.000	0.000
RED	14	1.000	0.000
REG	9	1.000	0.000
S144	6	1.000	0.000
V144	15	0.967	0.034
S139N	17	0.993	0.020
S139T	15	0.982	0.038
RRD	6	1.000	0.000
RGN	13	1.000	0.000
RGV	8	0.926	0.062
H146P	12	1.000	0.000
RGG	11	1.000	0.000
L147P	6	1.000	0.000
S139G	10	1.000	0.000
H146R	9	0.996	0.044
S139I	15	0.987	0.028
RGE1	11	0.927	0.040
**Total**	202	-	-
**Average**	11.882	0.987	0.016
**SD**	4.833	0.024	0.021

**Table 2:** Confusion matrix. Fraction of samples of each phenotype class classified in any other class (row-wise mode). Non zero values are highlighted in bold (see Methods).

**Genot.**	**RGD**	**RED**	**REG**	**S144**	**V144**	**S139N**	**S139T**	**RRD**	**RGN**	**RGV**	**H146P**	**RGG**	**L147P**	**S139G**	**H146R**	**S139I**	**RGE1**
**RGD**	**1.000**	0.000	0.000	0.000	0.000	0.000	0.000	0.000	0.000	0.000	0.000	0.000	0.000	0.000	0.000	0.000	0.000
**RED**	0.000	**1.000**	0.000	0.000	0.000	0.000	0.000	0.000	0.000	0.000	0.000	0.000	0.000	0.000	0.000	0.000	0.000
**REG**	0.000	0.000	**1.000**	0.000	0.000	0.000	0.000	0.000	0.000	0.000	0.000	0.000	0.000	0.000	0.000	0.000	0.000
**S144**	0.000	0.000	0.000	**1.000**	0.000	0.000	0.000	0.000	0.000	0.000	0.000	0.000	0.000	0.000	0.000	0.000	0.000
**V144**	0.000	**0.032**	0.000	0.000	**0.968**	0.000	0.000	0.000	0.000	0.000	0.000	0.000	0.000	0.000	0.000	0.000	0.000
**S139N**	**0.002**	0.000	0.000	0.000	0.000	**0.993**	**0.004**	0.000	0.000	0.000	0.000	0.000	0.000	0.000	0.000	**0.001**	0.000
**S139T**	0.000	0.000	0.000	0.000	0.000	**0.016**	**0.983**	0.000	**0.001**	0.000	0.000	0.000	0.000	0.000	0.000	0.000	0.000
**RRD**	0.000	0.000	0.000	0.000	0.000	0.000	0.000	**1.000**	0.000	0.000	0.000	0.000	0.000	0.000	0.000	0.000	0.000
**RGN**	0.000	0.000	0.000	0.000	0.000	0.000	0.000	0.000	**1.000**	0.000	0.000	0.000	0.000	0.000	0.000	0.000	0.000
**RGV**	**0.069**	0.000	0.000	0.000	0.000	0.000	0.000	0.000	0.000	**0.925**	0.000	**0.006**	0.000	0.000	0.000	0.000	0.000
**H146P**	0.000	0.000	0.000	0.000	0.000	0.000	0.000	0.000	0.000	0.000	**1.000**	0.000	0.000	0.000	0.000	0.000	0.000
**RGG**	0.000	0.000	0.000	0.000	0.000	0.000	0.000	0.000	0.000	0.000	0.000	**1.000**	0.000	0.000	0.000	0.000	0.000
**L147P**	0.000	0.000	0.000	0.000	0.000	0.000	0.000	0.000	0.000	0.000	0.000	0.000	**1.000**	0.000	0.000	0.000	0.000
**S139G**	0.000	0.000	0.000	0.000	0.000	0.000	0.000	0.000	0.000	0.000	0.000	0.000	0.000	**1.000**	0.000	0.000	0.000
**H146R**	0.000	0.000	0.000	0.000	0.000	0.000	0.000	0.000	0.000	0.000	**0.002**	**0.002**	0.000	0.000	**0.996**	0.000	0.000
**S139I**	**0.005**	0.000	0.000	0.000	0.000	**0.005**	**0.003**	0.000	0.000	0.000	0.000	0.000	0.000	0.000	0.000	**0.987**	0.000
**RGE1**	**0.001**	0.000	0.000	0.000	0.000	0.000	**0.068**	**0.002**	**0.002**	0.000	0.000	0.000	0.000	0.000	0.000	0.000	**0.927**

The raw data corresponding to this paper are provided as additional files ' [see Additional file1]' and can be found also in [28].

## Discussion

A microarray-based method to type representatives of the seven serotypes of FMDV has been developed by Baxi and colleagues [17]. The microarray contained 155 oligonucleotide probes, of 35 to 45 residues from the VP3-VP1-2A-coding region of the FMDV genome. We have now used a specific approach based on a microarray platform aimed at distinguishing point mutants within an important determinant of antigenicity and host cell tropism, namely the G-H loop of capsid protein VP1 (Figures 1 and 2). Several preliminary experiments showed a notorious decrease in the quality of results using aldehyde coated slides, streptavidine coated magnetic beads to obtain single-stranded DNA or a formamide hybridization solution. Additionally, other conditions involving nucleotide probes of different length, presence or absence of spacers between the array substrate and the probe, and different labeling and hybridization conditions were tested. The best signal to noise ratios and the most reproducible results were achieved using 15-mer with oligo (dT)_15 _spacer and 50 μM concentrated oligonucleotide probes, with the queried position located towards the center of the probe, printed of super-epoxi-coated slides (experimental conditions detailed in Methods). Hybridization and washing temperatures were also selected after systematic preliminary experiments.

To assess the reproducibility of the results, the classification accuracy was evaluated statistically using jack-knife simulations. This procedure revealed a high and stable degree of classification accuracy, although 2 variants were misclassified in more than 5% of cases. This was probably due to heterogeneity in the intensity of the hybridization reactions (Table 1 and 2). Despite this limitation in the reliable identification of some variants, the results illustrate the feasibility of a microarray approach to diagnose specific virus variants that may be associated with altered biological behaviors. Thus, the queried mutation was accurately discriminated from other mutations at the same site (Figure 4). In particular, the conserved L147 in VP1 is thought to be essential for integrin recognition of FMDV [29], and several substitutions at position 147 affect the interaction of FMDV of serotype C with antibodies. A variant with substitution L147P was isolated from a lesion of partially immunized cattle and had a profound effect on the antigenicity and tropism of FMDV [23]. This important L147P variant was correctly detected by the microarray. Crosshybridizations were observed with the probes to identify mutations that affect VP1 positions 142 and 143 (Figure 4b), expected from the high degree of overlap among these probes. This crosshybridization can be defined as the signal obtained when at least 9 nucleotides of a probe are perfect match with the target. For instance, mutant RGN shows a weak signal with the RGG probe, and the RGE mutant with the L144 probe. The two amino acids replaced in those variants are also essential for integrin recognition of FMDV [29].

Despite the bulk of microarray technology being used to define patterns of gene expression, increasing applications are found in the detection of genetic polymorphisms [30-32]. The application to discriminate among variants of FMDV is added to a number of microarray procedures used in virology to analyze multiple viral pathogens that belong to different virus families [18,33], to detect specific viruses [34-36] or to define genetic variations underwent by viruses [37,38] [reviews in [39,40]]. Microarray technology has been also used to probe differences in the structure of hepatitis C virus RNA, that result from genetic differences that may be associated with different responses to interferon treatment [41].

The distinction among mutants of the same virus is becoming increasingly necessary in view of the extensive variation among representatives of most virus groups [42], the quasispecies population structure of RNA viruses and some DNA viruses [8], and the increasing recognition that one or a limited number of mutations in a viral genome can have a profound effect in its biological behavior [reviews in [8,10,24]]. In this report, we have documented that DNA microarray technology can be used as a high-throughput method to analyze polymorphisms within a short region of the FMDV genome, and have successfully devised a SVM-based method to classify the samples on the basis of their hybridization signal. The procedure is of general applicability as a test for specificity and discriminatory power of microarray-based diagnostic procedures using multiple probes. We are currently investigating an extension of the same methodology to detect minority genomes in viral populations, as a means to quantify mutant spectrum complexity, and to evaluate memory levels in viral quasispecies [8,10,24].

## Conclusion

In the current study, we have documented that DNA microarray technology can be used as a high-throughput method to analyze polymorphisms within a short region of the FMDV genome encoding relevant functions in antigenicity and receptor recognition. We have successfully devised a support vector machine (SVM)-based method to classify the samples on the basis of their hybridization signal. The bioinformatic procedure is of general applicability to fine genotyping, including studies of heterogeneous viral populations, genetic changes in virus, bacteria, and genes of rapidly evolving cells, such as tumoral cells.

## Methods

### Cell culture and origin of FMDV mutants

Procedures for cell culture, infections with FMDV in liquid medium or in semisolid agar medium for titration of infectivity have been previously described [43]. FMDV biological clone C-S8c1, derived from natural isolate C-Sta Pau Sp/70 [43] was serially passaged 100 times in BHK-21 cells at a multiplicity of infection of 2–4 plaque-forming-units (PFU) per cell; this yielded population C-S8c1p100. Individual FMDV mutants with nucleotide substitutions at the genomic region encoding the G-H loop of VP1 were isolated by selecting escape mutants resistant to monoclonal antibody (mAb) SD6, which recognizes amino acids 138 to 147 of VP1 [44] (Figure 1). The populations used to select mAb SD6-resistant mutants were derived from C-S8c1p100, in experiments designed to test duration of quasispecies memory [45]. Procedures to select mAb SD6-resistant mutants were described previously [44,46,47].

### Microarray design and printing

Thirty eight DNA oligonucleotides, corresponding to the C-S8c1 genomic region encoding residues 139 to 147 (Figure 1) were designed and synthesized (Sigma). They included a 'C6 amino linker' [NH_2 _(CH_2_)_6_] at their 5'-end, followed by an oligo (dT)_15 _spacer and the specific 15-mer sequence; the oligonucleotides were purified by HPLC. The oligonucleotides (Figure 2) were selected to have a similar melting temperature when annealed to a complementary sequence, and included the queried nucleotide at the central region of the specific 15-mer. A conserved FMDV sequence, located between genomic residues 3757 and 3775 (5'-C6-T_15_CCTAGGCCGATTCTTCCG-3', within the VP1-coding region) [the numbering of FMDV genomic residues is according to [48]] was used as positive control for the hybridization (ICF, Internal Control FMDV). Two unrelated oligonucleotides (5'-C6-T_15_CAATACATGGATGATT-3' and 5'-C6-T_15_GATGCATATTTTTCAG-3', corresponding to the HIV reverse transcriptase coding region and termed HIVa and HIVb respectively) and spots with spotting solution with no nucleotide (nn in Figure 4) were used as negative controls. The oligonucleotides were diluted in 1 × spotting solution (Telechem-Arrayit) at 50 μM final concentration, and spotted onto super-epoxy-coated glass slides (Telechem-Arrayit).

Microarrays containing 384 spots were printed by means of a GMS 417 DNA arrayer (Affymetrix) defining four grids per slide. Each oligonucleotide was spotted in duplicate dots 150 μm in diameter, with a center-to-center distance of 250 μm (Figure 3).

In a number of preliminary assays, 11-mer and 15-mer oligonucleotides at concentrations of 5, 25 and 50 μM, and with or without an oligo (dT)_15 _spacer at the 5'-end were compared; the final protocol corresponds to the set of materials and conditions showing the highest sensitivity and reproducibility, among the conditions tested.

### Preparation of target DNAs

RNA from mAb SD6-escape mutants [45] was extracted using Trizol (Invitrogene), as previously described [48]. RNA was reverse transcribed using avian myeloblastosis reverse transcriptase (RT) and pUL as primer (5'-GAGAAGAAGAAGGCCCAGGGTG-3'; antisense primer, complementary to positions 3873 to 3896 of the FMDV C-S8c1 genome). PCR amplification of the cDNA was performed using Expand High Fidelity polymerase (Roche), as specified by the manufacturers; the primers used were 1R1L (5'-ACACCGTGTGTTGGCTACGGCG-3'; sense primer, corresponding to FMDV C-S8c1 genomic residues 3573 to 3594; phosphorylated at its 5'-end) and pUL. Each of the RT-PCR products was analyzed by nucleotide sequencing using the Big Dye Terminator Cycle Sequencing Kit (Abi Prism, Perkin Elmer) and the automated sequencers ABI 373 or ABI 3700, to ensure the presence of the mAb-escape mutation. The phosphorylated strand was specifically degraded using lambda exonuclease (New England Biolabs), and the resulting single-stranded DNA was labeled with Alexa Fluor 647 using the U-21660 Ulysis Nucleic Acid Labeling Kit (Molecular Probes). The labeled DNA was used as target in the hybridization with the probe oligonucleotides on the microarrays.

In a number of preliminary assays, a streptavidin-biotin system was assessed to obtain single-strand DNA target (AffiniTip Strep, -Hydros). Additionally, Cy3 and Cy5 fluorescence dyes (Amersham) were used as a direct labeling system. The final protocol includes the reagents showing in our hands the highest sensitivity and reproducibility.

### Hybridization and scanning

Immediately before hybridization, slides were processed as follows: They were washed for 2 min. at room temperature with 2X sodium saline citrate (SSC), 0.1% lauroylsarcosine, and for an additional 2 min. wash with 2 × SSC at room temperature, to remove unbound DNA and components of the printing buffer. The oligonucleotides were denatured by placing the slides 2 min. in distilled water at 100°C, cooled 10 sec. at room temperature, and then the oligonucleotides were fixed by plunging the slides into ice-cold 100% ethanol for 2 min., finally the slides were centrifuged 1 min. at 500 × g (Minicentrifuge Arrayit). Microarrays were incubated in a hybridization chamber (Genetix) with 20 μl of hybridization buffer (6 × SSC, 0.5% SDS, 1% BSA) under a 24 × 24 mm cover slip, and bathed at 42°C for 45 min. Then the microarrays were washed with distilled water, and dried by a brief centrifugation.

The hybridization with the labeled DNA was carried out in hybridization buffer at the appropriate temperature (58–60°C) and with the required amount of target (0.3 pmoles Alexa Fluor 647 equivalent to 50 ng). After a 3 hours incubation in the hybridization chamber, the slides were washed for 5 min. in 2 × SSC, 0.1% lauroylsarcosine, followed by 5 min. in 2 × SSC, and finally rinsed 10 sec. in 0.2 × SSC, and 5 min. in distilled water, at 45°C. The slides were dried by spinning 1 min. at 500 × g and, finally, scanned using a G2565AA/G2565AB Scanner (Agilent). The Agilent and Scan Array Express (Perkin Elmer Life Sciences) analysis software was used for reading and quantifying the hybridization images. The reproducibility of the method was assessed by comparing the results of at least five different hybridization experiments for each mutant.

### Data pre-processing

Array quantification was performed with the program Scan Array Express. Each probe was duplicated in the array. For each spot in the array, measures for the mean and median foreground intensity and for the mean and median background intensities were available. Visual inspection of scanned hybridized arrays revealed some noise due to the presence of dust and scratches, introducing an uneven increase in the mean foreground signal for some spots. We have tried to detect the affected spots by calculating a Z-score of their mean foreground intensity per pixel, using the four measurements available for each probe in every hybridization experiment. For this, we have used the additional measures available for the same probe (median foreground intensity and replicated spot mean and median foreground intensity) and computed their average and standard deviation. Then we calculated the Z-score in the usual way, subtracting the average from the mean foreground intensity and then dividing it by the standard deviation. After testing several absolute Z-score thresholds for discarding spots, we have found that a Z-score of 7 provides optimal results. If neither of the spots is discarded, we take as a measure for the presence of each mutant in the sample (M_*a*_), the log_2 _of the average of its replicated spots mean foreground intensities, subtracted from its background:

Ma=log⁡2(Ia1−Ba1+Ia2−Ba22)
 MathType@MTEF@5@5@+=feaafiart1ev1aaatCvAUfKttLearuWrP9MDH5MBPbIqV92AaeXatLxBI9gBaebbnrfifHhDYfgasaacH8akY=wiFfYdH8Gipec8Eeeu0xXdbba9frFj0=OqFfea0dXdd9vqai=hGuQ8kuc9pgc9s8qqaq=dirpe0xb9q8qiLsFr0=vr0=vr0dc8meaabaqaciaacaGaaeqabaqabeGadaaakeaacqWGnbqtdaWgaaWcbaGaemyyaegabeaakiabg2da9iGbcYgaSjabc+gaVjabcEgaNnaaBaaaleaacqaIYaGmaeqaaOGaeiikaGYaaSaaaeaacqWGjbqsdaWgaaWcbaGaemyyaeMaeGymaedabeaakiabgkHiTiabdkeacnaaBaaaleaacqWGHbqycqaIXaqmaeqaaOGaey4kaSIaemysaK0aaSbaaSqaaiabdggaHjabikdaYaqabaGccqGHsislcqWGcbGqdaWgaaWcbaGaemyyaeMaeGOmaidabeaaaOqaaiabikdaYaaacqGGPaqkaaa@4924@

where I_*a*1 _and I_*a*2 _are the mean foreground pixel intensities and B_*a*1 _and B_*a*2 _are the mean background pixel intensities for spot 1 and spot 2 of the probe for variant *a*, respectively. In case one of the spots is discarded, we take M_*a *_as the log_2 _of the remaining spot mean foreground intensity subtracted from its mean background intensity.

As a hybridization quality control, we added a probe for a fully conserved region of VP1 of the FMDV (ICF), discarding those arrays for which the log_2 _of the average intensity for this probe was under 7, in our experience a threshold that distinguishes arrays with hybridization problems from the normal ones. We tried several normalization conditions as taking the square root of the average spots mean intensities instead of the log_2 _or making a prior normalization by dividing each M_*a *_by M_*ICF*_, but final classification accuracy was optimal at the conditions reported.

### Data classification

Data classification was carried out with a multiple class support vector machine tool (mcSVM) [49,50]. Briefly, a SVM is a supervised learning algorithm [51]. It belongs to the class of methods that solve the general problem of learning discriminative boundaries, able to optimally separate positive and negative members of a given set of points in a *n*-dimensional vector space. The SVM algorithm operates by first mapping the training set into a high-dimensional feature space and then attempting to locate in that space a hyperplane that separates positive from negative examples. Having found such a hyperplane, the SVM can then predict the classification of an unlabeled example by mapping it into the feature space and asking on which side of the separating plane the example is found. The multiclass SVM is an extension of the classification problem to multiple classes, instead of just a binary classification.

In our case, we have used 39 probes in the array for classification purposes, one for quality control ICF and 38 for detecting different genotypes, including mutants and wild type. Therefore, each sample was encoded by a 39 dimensional vector, each dimension corresponding to a variable computed in equation 1. We analyzed 202 samples distributed among 17 phenotype classes to classify (Table 1). We ensured that at least 6 samples were available for each variant (Table 1). We applied mcSVM to this problem, using a Gaussian kernel which yielded γ = 10^-2 ^and α = 10^3 ^as optimal parameters.

### Assessing the classifier

In order to test the prediction capabilities of the method, we applied a jack-knife test. We assigned randomly the samples to 10 different groups. Each one of the groups, with 10% of the samples, was used as a test set, while the remaining 90% was used as a training set. We then measured the fraction of correctly predicted samples by mcSVM in the test. The procedure was repeated for all groups, completing in this way one round of testing. 100 rounds were simulated, each time with a different random distribution of samples in the groups. We averaged out the fraction of correctly predicted samples to obtain the final quality of the classifier. We also built a confusion table in order to study the presence of systematic errors in the cases that failed (Table 2). This table shows in a row-wise mode the fraction of samples of each phenotype variant classified in any other variants.

## Authors' contributions

VM and CP performed most of the experiments, have been involved in conception and design of the study, in target preparation, acquisition, analysis and interpretation of data, and helped to prepare the manuscript.

DA and ARO performed the bioinformatics analysis and contributed to interpretation of the data and the writing of the manuscript.

ED conceived and designed the study, had FMDV mutants to prepare the targets, drafted the manuscript, and revised it critically for important intellectual content.

CB conceived the study, set up DNA microarrays technology for this approach and printed the arrays, in the design and coordination of experiments and helped to prepare the manuscript.

All authors read and approved the final manuscript.

## Supplementary Material

Additional File 1 They are obtained using the Agilent and Scan Array Express (Perkin Elmer Life Sciences) analysis software for reading and quantifying the hybridization images. Click here for file
